# Frequency of Cardiovascular Involvement in Familial Amyloidotic Polyneuropathy in Brazilian Patients

**DOI:** 10.5935/abc.20150112

**Published:** 2015-11

**Authors:** Márcia Cavalcanti de Campos Queiroz, Roberto Coury Pedrosa, Amanda Cardoso Berensztejn, Basílio de Bragança Pereira, Emília Matos do Nascimento, Martha Maria Turano Duarte, Pedro Paulo Pereira-Junior, Marcia Waddington Cruz

**Affiliations:** 1 Universidade Federal do Rio de Janeiro - Instituto do Coração Edson Saad, Rio de Janeiro, RJ - Brazil; 2 Hospital Naval Marcílio Dias - Marinha do Brasil, Rio de Janeiro, RJ - Brazil; 3 Universidade Federal do Rio de Janeiro - Instituto Alberto Luiz Coimbra de Pós-Graduação e Pesquisa em Engenharia, Rio de Janeiro, RJ - Brazil; 4 Universidade Federal do Rio de Janeiro - Instituto de Biofísica, Rio de Janeiro, RJ - Brazil; 5 Universidade Federal do Rio de Janeiro - Centro de Estudos em Paramiloidose Antônio Rodrigues de Mello, Rio de Janeiro, RJ - Brazil

**Keywords:** Amyloid Neuropathies, Familial, Cardiomyopathies, Prealbumin, Eletrocardiography

## Abstract

**Background:**

Familial amyloidotic polyneuropathy (FAP) is a rare disease diagnosed in Brazil and worldwide. The frequency of cardiovascular involvement in Brazilian FAP patients is unknown.

**Objective:**

Detect the frequency of cardiovascular involvement and correlate the cardiovascular findings with the modified polyneuropathy disability (PND) score.

**Methods:**

In a national reference center, 51 patients were evaluated with clinical examination, electrocardiography (ECG), echocardiography (ECHO), and 24-hour Holter. Patients were classified according to the modified PND score and divided into groups: PND 0, PND I, PND II, and PND > II (which included PND IIIa, IIIb, and IV). We chose the classification tree as the statistical method to analyze the association between findings in cardiac tests with the neurological classification (PND).

**Results:**

ECG abnormalities were present in almost 2/3 of the FAP patients, whereas ECHO abnormalities occurred in around 1/3 of them. All patients with abnormal ECHO also had abnormal ECG, but the opposite did not apply. The classification tree identified ECG and ECHO as relevant variables (p < 0.001 and p = 0.08, respectively). The probability of a patient to be allocated to the PND 0 group when having a normal ECG was over 80%. When both ECG and ECHO were abnormal, this probability was null.

**Conclusions:**

Brazilian patients with FAP have frequent ECG abnormalities. ECG is an appropriate test to discriminate asymptomatic carriers of the mutation from those who develop the disease, whereas ECHO contributes to this discrimination.

## Introduction

Familial amyloidotic polyneuropathy (FAP), also called hereditary transthyretin-related amyloidosis (ATTR), is a rare disease diagnosed in Brazil and worldwide. The prevalence of FAP is unknown in Brazil, but the disease has been determined to affect 1 out of 1000 individuals in Portugal^1^. This autosomal dominant disease is caused by a mutation in the transthyretin (*TTR*) gene located on chromosome 18.The most common mutation, Val30Met, is also the most commom mutation that affects FAP patients in Portugal. Considering that Brazil was colonized by Portugal, this may also be the predominant mutation in our country.

FAP is typically manifested by a progressive sensory-motor polyneuropathy, often with severe autonomic neuropathy^2^. Cardiovascular involvement may also occur in the disease, although the frequency of this involvement in Brazilian patients is unknown.

We addressed two interrelated questions in this study: 1) what is the frequency of cardiovascular involvement in Brazilian FAP patients with the Val30Met mutation? 2) is there a relationship between cardiovascular involvement and the modified polyneuropathy disability (PND) score?

## Methods

This study included 51 patients attending the *Centro de Estudos em Paramiloidose Antônio Rodrigues de Mello* (CEPARM), a Brazilian national reference center at the Federal University of Rio de Janeiro (Brazil), from October 2012 to September 2013. All patients were at least 20 years old and had a mutation in the *TTR* gene. They all underwent a complete clinical examination by the same physician on the first visit, with special attention to signs and symptoms of cardiovascular disease (syncope or near-syncope, palpitations, thoracic pain, and heart failure according to the European Society of Cardiology [ESC] criteria^3^), electrocardiography (ECG), echocardiography (ECHO), and 24-hour Holter. We excluded patients with evidence of other heart problems, pregnancy, previous treatment with drugs that might cause any cardiovascular side effects, severe renal dysfunction, and alcoholism. The presence of systemic arterial hypertension was not an exclusion criterion, unless left ventricular (LV) hypertrophy was evident on the ECG.

The study complied with the Declaration of Helsinki and had its protocol approved by the local ethics committee. All patients signed a written informed consent form.

Patients were classified according to the modified PND score^4^: 0 - no sensory disturbances; I - sensory disturbances in the feet, but no impaired walking capability; II - walking impairment, but walking without aid; IIIa - walking with one stick or crutch; IIIb - walking with two sticks or crutches; and IV - confined to a wheelchair or bedridden. According to the classification, patients were divided into four groups: PND 0, PND I, PND II, and PND >II (this last one included patients with PND IIIa, IIIb, and IV).

The ECGs were interpreted according to the Minnesota code^5^ and the ECHOs according to the recommendations of the American Society of Echocardiography^6-9^. A result was considered abnormal when at least one of the following criteria were described: on ECG - a non-sinus rhythm, atrioventricular (AV) or intraventricular block, low voltage and/or abnormal ventricular repolarization; on ECHO - end-diastolic thickness of the interventricular septum > 12 mm, AV valve thickening, increased myocardial echogenicity, and/or systolic or diastolic dysfunction; and on 24-hour Holter - atrial tachyarrhythmia, ventricular tachycardia, AV block, supraventricular ectopic beats in moderate or high incidence, and/or ventricular ectopic beats in moderate or high incidence. Low voltage was defined as a QRS voltage amplitude < 0.5 mV in all limb leads, and < 1 mV in all precordial leads.

Amyloidotic cardiomyopathy was diagnosed by a non‑cardiac positive biopsy associated with echocardiographic evidence of amyloidosis^10^. This last was characterized by the same criteria used by Trikas et al^11^, namely, presence of mean LV wall thickness > 12 mm and 2 or more of the following criteria: homogeneous valve thickening, atrial septal thickening, sparkling appearance of the ventricular septum, and restrictive LV function. Cardiac involvement was defined by an abnormal result on at least one of the tests (ECG, ECHO, or 24-hour Holter).

We chose the classification tree as the statistical method to analyze the association between abnormalities in cardiovascular tests (ECG, ECHO, and 24-hour Holter) with the neurological classification (PND). The classification tree uses the CART method, an algorithm of recursive partitioning^12^. The Party package of the R software was used for the analysis^13^. A p value < 0.05 was considered significant.

## Results

**Figure 1:**
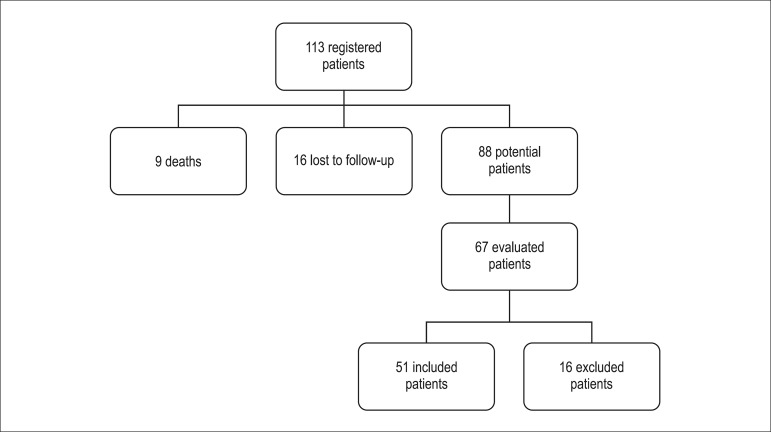
Organogram showing the patients’ flow in the process of evaluation and selection for the study.

A total of 67 patients were evaluated. Of these, 16 were excluded for different reasons: alcoholism (1), pregnancy (1), death before completion of the tests (1), mutation not confirmed (2), previous radiotherapy (1), and use of goserelin acetate, a medication that can cause heart failure as side effect (1). Nine were excluded for not completing the cardiovascular evaluation. The final analysis included 51 patients (Figure 1) with a mean age of 36.1 years at the diagnosis of the disease.

The general characteristics of the patients, including comorbidities, are presented in Table 1. There were six hypertensive patients included, two of whom had normal ECHOs, and three had mild diastolic dysfunction (level 1) without evidence of hypertrophy on ECG or ECHO, or other target organ injury. One patient who had mild hypertension and blood pressure levels around the normal range even in the absence of medication had significant increase in the myocardial wall thickness with increased echogenicity, a characteristic of infiltrative disease.

**Table 1:** Patients' characteristics and comorbidities

	N	(%)
Total number of patients studied	51	(100%)
Age - Mean ± SD (years)	40.27±11.48	-------
Male gender	25	49%
Val30Met mutation	48	94.1%
Liver transplantation	10	19.6%
Use of tafamidis	5	9.8%
PND 0	21	41%
Diabetes mellitus	1	1.96%
Hypertension	6	11.8%
Chronic renal failure	2	3.9%
Smoking	4	7.84%
Caucasian	43	84.3%

PND: Polyneuropathy disability score.

**Figure 2:**
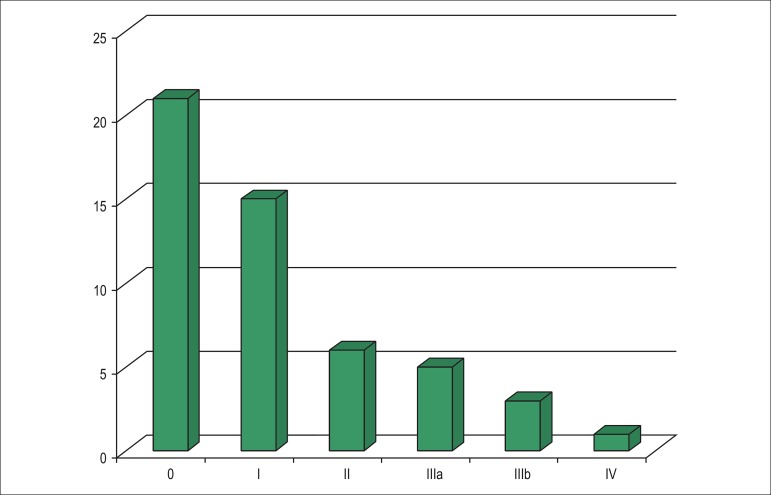
Patients’ distribution according to the PND score.

Figure 2 shows the distribution of the patients according to the neurological classification (PND score). Most patients (41%) had a PND score of 0.

Five patients (9.8%) were diagnosed with amyloidotic cardiomyopathy. The prevalence of heart failure in the studied population was 2% (one patient). It is important to emphasize that all patients diagnosed with amyloidotic cardiomyopathy also had neurological impairment (PND ≥ I).

The abnormalities observed in the cardiac tests are shown in Table 2. ECG abnormalities affected 2/3 of the total cohort of Brazilian patients with FAP, whereas changes in ECHO occurred in around 1/3 of them. Although all patients with abnormal ECHO also had an abnormal ECG, the opposite did not apply.

**Table 2:** Frequency of abnormalities in cardiac tests in the studied patients

	Number of Patients	%
**ELECTROCARDIOGRAPHY**		
Atrioventricular conduction disturbance	7	13.7
Intraventricular conduction disturbance	10	19.6
Low QRS voltage	1	1.96
Inespecific ventricular repolarization alterations	17	33.3
Non-sinus rhythm	4	7.8
Abnormal	34	66.7
**24-HOUR HOLTER**		
Atrial tachycardia	7	13.7
Supraventricular ectopic beats of moderate frequency	2	3.9
Supraventricular ectopic beats of high frequency	4	7.8
Nonsustained ventricular tachycardia	5	9.8
Ventricular ectopic beats of moderate frequency	3	5.9
Ventricular ectopic beats of high frequency	2	3.9
Abnormal	20	39.2
**ECHOCARDIOGRAPHY**		
Increased ventricular wall thickness > 12 mm	7	13.7
Increased myocardial echogenicity	10	19.6
Homogeneus valve thickening	12	23.5
Diastolic dysfunction level I	12	23.5
Diastolic dysfunction level II	3	5.9
Diastolic dysfunction level III	1	1.96
Diastolic dysfunction level IV	0	0
Abnormal	19	37.2

### Association between the neurological classification (PND) and results in the cardiovascular tests

**Figure 3:**
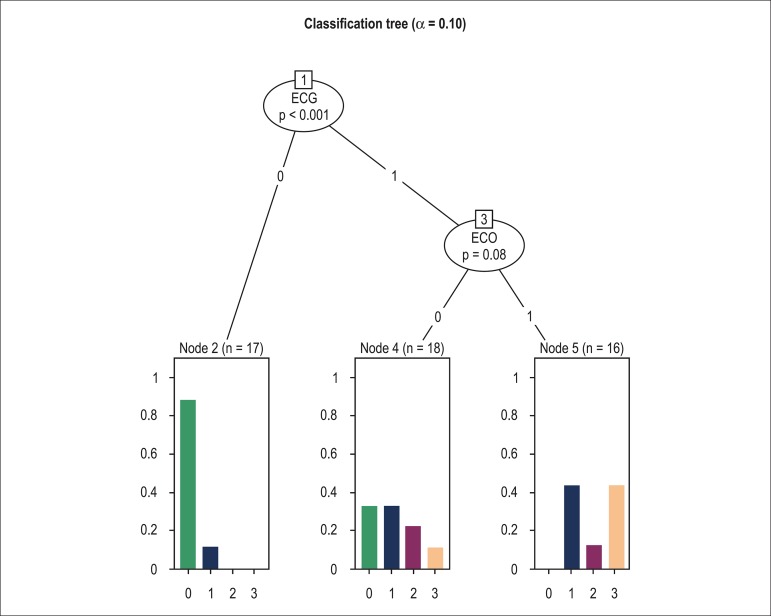
Classification tree analysis of the association between results in the cardiovascular tests with the neurological classification. ECG 0 = Normal ECG, ECG 1 = Abnormal ECG, ECO 0 = Normal echocardiogram, ECHO 1 = Abnormal echocardiogram. In the abscissa axis, 0 = PND 0, 1 = PND I, 2 = PND II, 3 = PND > II.

The classification tree (Figure 3) identified the ECG as the main variable discriminating patients according to the neurological classification PND (p < 0.001). In the subgroup with abnormal ECG, ECHO (p = 0.08) was an additional variable discriminating patients according to the PND score. In the presence of normal ECG (ECG = 0), the probability of a patient to be placed in the PND 0 group was over 80%. When both ECG and ECHO were abnormal (ECG = 1 and ECHO = 1), the probability of a patient to be placed in the PND 0 group was null. The classification tree did not identify the 24-hour Holter as relevant, even when alpha was set at 20% (cut point).

## Discussion

This study evaluated Brazilian patients with FAP followed at CEPARM, including asymptomatic carriers of the disease mutation and patients with manifestations of the disease. The results present strong evidence that ECG is an appropriate test to discriminate asymptomatic carriers of the mutation from those who have developed the disease. This was demonstrated by the classification tree, which identified the relevance of ECG (in the presence of a normal ECG, the probability of a patient to receive a PND score of 0 was over 80%). ECHO (p = 0.08) was an additional variable that helped discriminate patients in the subgroup with abnormal ECG according to the modified PND score (when both ECG and ECHO were abnormal, the probability of a patient to be placed in the PND 0 group was null).

The classification tree did not detect the 24-hour Holter as a relevant variable even when alpha was set at 20%. Therefore, the 24-hour Holter is not an appropriate test to discriminate the groups, probably due to the low frequency of arrhythmias.

Two other studies using ECG to assess the cardiac involvement in FAP reported abnormal findings. Rapezzi et al analyzed 61 patients with ATTR (11 patients with the Val30Met mutation) and found that 90% of them had abnormal ECGs. Koike et al^15^ performed a study in Japan to elucidate the natural history of the Val30Met mutation in patients with late-onset FAP in non-endemic areas. An AV conduction disturbance (first and second degree AV block) was detected in five out of 41 (13%) patients examined.

In the present study, 66.7% of the patients had abnormal ECG. In a systematic review to evaluate the pattern and incidence of cardiovascular involvement in FAP in Portugal, Freitas^16^ found 87.2% of abnormal ECGs. Data from both these studies are not comparable, since the selected populations had different characteristics. Freitas^16^ included patients with typical neurological picture and a positive cutaneous or nerve biopsy, whereas our study also included asymptomatic carriers of the mutation.

When we analyzed the ECG abnormalities, we observed that four patients (7.8%) with a non-sinus rhythm had a paced rhythm. This frequency is similar to the one observed by Rahman et al^17^ who found a paced rhythm in 8% of the patients with cardiac amyloidosis diagnosed by biopsy.

Only one patient (1.96%) had a low QRS voltage. It is important to mention that we considered the occurrence of a low voltage when the criteria was present in the limb and precordial leads. O'Donnell et al^18^ found a low QRS voltage in 4% of the patients with hereditary amyloidosis, but different from the present study, these authors evaluated patients with proven cardiac amyloidosis.

An AV conduction disturbance was present in 13.7% of the patients. O'Donnell et al^18^ found a prevalence of 40% of AV conduction disturbance in patients with hereditary amyloidosis. However, as previously mentioned, the selected populations had different characteristics.

Although the use of ECG is common in publications about FAP, its use as a strategy to associate the occurrence of cardiac involvement with the degree of severity of neurological symptoms expressed by PND has never been reported. Our main finding was that asymptomatic carriers of the Val30Met mutation (PND 0) may show an early sign of cardiac involvement, demonstrated by the presence of unspecific ventricular repolarization abnormalities in 33% of the cases. This may correspond to an incipient abnormality in the process of ventricular repolarization caused by amyloid infiltration.

As in other cardiac diseases, ECG has been consistently demonstrated as the main marker of cardiac involvement in FAP^18^_,_^19^, which emphasizes the importance of its noninvasive stratification. Unfortunately, two-dimensional echocardiography is not available in many Brazilian areas, and the evaluation of the cardiac involvement must then rely on clinical, radiological, and ECG grounds, pointing out the relevance of this study findings.

In addition, as shown in the patients in the present study, a normal ECG virtually excludes the possibility of significant cardiac involvement (in fact, ECHO was only an additional variable in discriminating patients according to PND score in the subgroup with abnormal ECG). However, FAP patients with clinically overt congestive heart failure have the worst prognosis. In the present study, heart failure was uncommon (only one patient). Thus, the subgroup of patients in whom it is most relevant to stratify the cardiac involvement is that with abnormal ECG and without clinical heart failure. In this particular group, the classification tree identified the relevance of the ECG as a relatively simple, noninvasive, inexpensive, and readily available test.

It is important to highlight that even unspecific changes in ventricular repolarization were considered abnormal. This is usually considered a variation of the normal. Thus, when a normal ECG is said to discard significant cardiac involvement, it is not the absence of the pathognomonic low QRS voltage that is under consideration, but rather, the absence of even unspecific ventricular repolarization abnormalities. Considering areas in which ECHO is not broadly available, this information is of great interest.

For practical applications, we suggest that all FAP patients should have a complete clinical examination and an ECG on the first medical workup. Patients with overt decompensated heart failure and evident LV dysfunction should be treated accordingly, and those with normal ECGs should be considered as having no significant cardiac involvement and only be followed up with annual ECGs. Those patients without clinically decompensated heart failure and with abnormal ECGs should be evaluated with ECHO. If the ECHO is normal, these patients should be considered as having a low probability of presenting a myocardial involvement and be followed up every six months. If the ECHO is abnormal, depending on the abnormalities found, the patient should be regarded as having amyloidotic cardiomyopathy, and then treated and followed up closely.

The large number of patients with PND 0 (41%) can be explained by the fact that most family members of the patients at CEPARM express willingness to undergo genetic study and, when the result is positive, continue to be followed up.

In the studied group, there were 10 patients who had already undergone liver transplantation. We considered the possibility that the cardiovascular changes were caused by immunosuppressive agents. Only atrial and ventricular arrhythmias may be side effects of immunosuppressive agents, but since the 24-hour Holter was not identified as a relevant variable by the classification tree, this did not interfere with the study's results.

One limitation of our study is the absence of measurement of biomarkers in the evaluation of the cardiovascular involvement in FAP patients. However, measurement of biomarkers is not broadly available in the public health services in our country. Another limitation is the small number of patients, but considering the rarity of this disease (1/1000 individuals in Portugal, that is an endemic area), the fact that CEPARM is a national reference center, and that many studies about FAP include less than 50 patients, we believe that this study contributes to the knowledge of this disease in our country.

## Conclusions

Brazilian patients with FAP have frequent ECG abnormalities. ECG emerged as an appropriate test to discriminate asymptomatic carriers of the mutation from those who have developed the disease, whereas ECHO contributed to this discrimination.
